# Understanding embryonic development at single-cell resolution

**DOI:** 10.1186/s13619-020-00074-0

**Published:** 2021-01-27

**Authors:** Mengmeng Jiang, Xue Xu, Guoji Guo

**Affiliations:** 1 Center for Stem Cell and Regenerative Medicine, Zhejiang University School of Medicine, Hangzhou, 310058 China; 2 Zhejiang University Medical Center, Hangzhou, 310058 China; 3 Wuhan University of Science and Technology, School of Medicine, Wuhan, 430000 China; 4 Bone Marrow Transplantation Center, The First Affiliated Hospital, Zhejiang University School of Medicine, Hangzhou, 310009 China; 5 Institute of Hematology, Zhejiang University, Hangzhou, 310058 China

A major goal of developmental biology is to understand the progression of embryonic cell lineages from pluripotency to adulthood.  During embryogenesis, cells proliferate and spread out, new cell types arise, while many progenitors fade away during differentiation. Early studies of cell-fate regulation and lineage tracing have enriched out knowledge about the developmental processes. Yet, a complete picture about the spatiotemporal cellular composition of the developing embryo and the cellular interactions coordinating embryonic development is lacked for most of the model systems that we study.

Individual cells are fundamental units of animal development. Understanding heterogeneity between and within cell types is important for comprehending cellular physiology and manipulating biological systems (Regev et al. 2017). Traditional bulk tissue sequencing can only measure the averages across a population of cells or complex tissues, and therefore lose the  information about cellular heterogeneity. Fortunately, recent progress in single cell profiling technologies has revolutionized our ability to identify cellular composition and track molecular dynamics in developmental biology. In 2009, Tang et al. first reported the study of mRNA transcriptome sequencing from a single cell. In 2012, Smart-seq, which detects full-length transcripts in single cells has been developed. Since 2015, with the continuous advent of Fluidigm C1, Drop-seq, inDrop, 10× Genomics, Seq-well, Microwell-seq, Split-seq and other technologies, single-cell researches have completely entered the era of high-throughput, low-cost and automation (Stark et al. 2019). Accompanied by the breakthroughs in technologies, the development of computational methods empowered our ability to define developmental cell states and infer developmental trajectories at single-cell resolution  (Farrell et al. 2018).

Our understanding of human development has been largely inferred from studies on organoid model and culture systems. However, a comprehensive reference atlas of cell types and states present during embryonic development will be critical to show in vivo biological processes. Transcriptomes of 1529 individual cells from 88 human preimplantation embryos were profiled, which showed lineage segregation of trophectoderm, primitive endoderm, and pluripotent epiblast (Petropoulos et al. 2016). Novel genes (*ARGFX*, *LINC00261*) were identified, which might be important for human preimplantation development. Using three-level single-cell combinatorial indexing for gene expression (sci-RNAseq3), Cao et al. constructed a comprehensive human cell atlas by profiling gene expression of 121 fetal samples ranging from 72 to 129 days in estimated post-conceptual age, altogether representing 4 million single cells. In this tremendous gene expression molecular atlas, novel cell types were identified and validated in unexpected tissues, such as potentially circulating trophoblast-like and hepatoblast-like cells (Cao et al. 2020). These single-cell data resource bridges gene expression dynamics from the embryonic to the fetal stages of human development. Our group extended and deepened the human cell landscape (HCL) by analyzing fetal-to-adult cell-type transitions. We found that stem and progenitor cells were transcriptionally indistinct and stochastic, while differentiated cells were transcriptionally distinct and stable (Han et al. 2020).

Unlike human, mice develop quickly, with just 3 weeks  between fertilization and birth. At the early stages, the embryo transits from gastrulation to early organogenesis (E6.5–E8.5). In the ensuing days, the embryo amplifies to over ten-million cells rapidly and develops nearly all major organ systems (E9.5–E13.5). Pijuan-Sala et al. profiled transcriptomes of mouse embryos from E6.5 to E8.5, which explored developmental trajectories from gastrulation to early organogenesis. In addition, mutated *Tal1* was found to display defects in early mesoderm diversification (Pijuan-Sala et al. 2019). Cao et al. profiled the transcriptomes of around 2 million cells staged between 9.5 and 13.5 days of gestation using sci-RNA-seq3, providing a global view of developmental processes during mouse organogenesis (Cao et al. 2019b). The dynamics of gene expression within hundreds of cell types and trajectories over time were explored in the apical ectodermal ridge, limb mesenchyme and skeletal muscle. Fate-mapping and lineage-tracing studies of spatiotemporal transcriptome are key process during vertebrate embryogenesis. A recent interesting development is the spatially resolved transcriptome of cell populations at defined positions during mouse embryogenesis, further enriching the content of the mouse developmental cell atlas (Peng et al. 2019). Single-cell transcriptomics offers the opportunity to compare cell types across species. In comparative analysis of HCL and mouse cell atlas data, we found that cell-type similarity in orthologous gene expression overrides species differences (Han et al. 2020). Similarly, by integrating four million human fetal data with two million mouse embryonic datasets, single cell cross-species analyses are expected to indeed reveal conserved and divergent transcriptional programs during embryonic development (Cao et al. 2019b; Cao et al. 2020). We envision broad utility of transcriptional atlas in future studies on mammalian development research.

Single-cell expression profiling surveys of cellular differentiation hierarchies promise to empower systematic interrogations of developmental biology. Studies of zebrafish whole-embryo developmental landscapes described individual cell states transitioning from pluripotent blastomeres to a large array of cell types and tissues. Zebrafish embryogenesis single-cell lineage histories were reconstructed by developing a transposon-based barcoding approach (Wagner et al. 2018). Coincidentally, Farrell et al. sequenced 38,731 single cells and reconstructed transcriptional trajectories of zebrafish embryo using URD, a simulated diffusion-based computational reconstruction method (Farrell et al. 2018). Briggs et al. profiled 136,966 single-cell transcriptomes over the first day of life of *Xenopus*, and constructed a map of differentiation across all lineage over time. Studies revealed many embryonic cell states appeared earlier than previously appreciated (Briggs et al. 2018)*.* In addition to vertebrates, Table 1 also showed other species transcriptional profiles of embryonic development at single-cell level, such as *Drosophila*, *C. elegant* and *Ciona intestinalis* (Cao et al. 2019a; Karaiskos et al. 2017; Packer et al. 2019).

**Table 1:** Studies of embryonic development at single-cell level

Species	Stage	Method	Cell number	Reference
Human	E3-E7	Smart-seq2	1529	Petropoulos et al. 2016
Human	E72-E129	sci-RNA-seq3	4 million	Cao et al. 2020
Mouse	E6.5-E8.5	10× Genomics	116,312	Pijuan-Sala et al. 2019
Mouse	E9.5-E13.5	sci-RNA-seq3	2 million	Cao et al. 2019b
Zebrafish	3.3hpf-12hpf	Drop-seq	38,731	Farrell et al. 2018
Zebrafish	4hpf-24hpf	in-Drop	92,000	Wagner et al. 2018
*Xenopus*	5hpf-24hpf	Drop-seq	136,966	Briggs et al. 2018
*Drosophila*	stage 6	Drop-seq	7975	Karaiskos et al. 2017
*C. elegans*	embryo	10× Genomics	86,024	Packer et al. 2019
Ascidian	embryo	10× Genomics	90,000	Cao et al. 2019a

Collectively, these single-cell transcriptome analyses described embryonic gene expression with unparalleled temporal and cellular resolution. Reconstructed complex developmental trees illuminated system level lineage trajectory and cell fate decision across an entire organism, providing an atlas of the expression pattern and dynamics for nearly all genes. Continuously evolving computation analysis tools are expected to precisely infer spatiotemporal progressions and the regulatory programs in developmental systems at single-cell level (Farrell et al. 2018; Karaiskos et al. 2017). This allows inspection of lineage gene co-expression easy, reveals markers of cell types of interest, associates characterized genes with cell fate specification, and eventually empowers our understanding of embryonic development. Besides, molecular processes that guide embryonic development can be further comprehended by studying developmental disorders. Single-cell profiling for mutant embryos identified gene function defects in developmental stages (Pijuan-Sala et al. 2019; Wagner et al. 2018). Finally, integrated single-cell developmental profiles for different species also enable comparative studies, which will promote revealing conserved pathways, species-specific idiosyncracies and tracing the evolutionary origin of cell types (Briggs et al. 2018; Cao et al. 2019a).

  In conclusion, large-scale single-cell transcriptome profiles of embryonic development provide useful resources for us to understand organogenesis, cell fate decisions, and the molecular basis of tissue regeneration. We anticipate significant progresses will be made in the coming years to build a multi-species developmental roadmaps for the research communities. 
